# Evaluation of Cardiac Structural Changes Induced by Carbamazepine-Based Nanotherapeutics in an Experimental Epilepsy Model

**DOI:** 10.3390/nano15221732

**Published:** 2025-11-17

**Authors:** Adem Tokpınar, Hasan İlhan, Semih Tan, Selen Kazancı, Cemre Zeynep Harman Civek, Rabia Kurt Tokpınar, Emin Kaymak, Muhammet Değermenci, Orhan Baş

**Affiliations:** 1 Department of Anatomy, Faculty of Medicine, Ordu University, Ordu 52000, Türkiye; selenylmz.sln@gmail.com (S.K.); mdegermenci@yahoo.com.tr (M.D.); orhanbas55@hotmail.com (O.B.); 2 Department of Biotechnology, Institute of Biotechnology, Ankara University, Ankara 06110, Türkiye; 3 Department of Histology-Embriology, Faculty of Medicine, Ordu University, Ordu 52000, Türkiye; tansemih@hotmail.com (S.T.); czharman@hotmail.com (C.Z.H.C.); 4 Department of Physiology, Faculty of Medicine, Erciyes University, Kayseri 38280, Türkiye; rabiakurttokpinar@gmail.com; 5 Department of Histology-Embriology, Faculty of Medicine, Yozgat Bozok University, Yozgat 66100, Türkiye; e_kaymak@hotmail.com

**Keywords:** epilepsy, carbamazepine, carbon nanodots, heart morphology, metal-organic frameworks (MOF-5)

## Abstract

**Background/Objectives**: This study was conducted to investigate the morphological impact of carbamazepine (CBZ) coated with carbon nanodots functionalised with silver nanoparticles (CNDs@AgNPs) and metal–organic framework (MOF-5) nanoparticles on the hearts of male rats with experimental epilepsy. **Methods**: Seventy male Wistar rats were randomly selected for the study and divided into ten groups of seven animals each. Haematoxylin–eosin staining was performed on heart tissue, and the levels of interleu-kin-6 (IL-6) and catalase (CAT) and the oxidative stress index (OSI) were determined bio-chemically. In addition, we performed morphological measurements of the heart. **Results**: When the heart tissues were evaluated histopathologically in all groups, it was observed that cells with pyknotic nuclei and haemorrhagic areas increased in the heart images, especially in the PTZ group with epilepsy only. Histologically normal cardiac cells and cardiac tissue were observed in the other groups. The distance between the atria was below 10 mm only in PTZ + CBZ 50 mg/kg and PTZ + CNDs@MOF-5 25 mg/kg groups. The distance between the apex of the heart and the base of the heart was the lowest in CNDs@MOF-5 25 mg/kg and CNDs@MOF-5 50 mg/kg groups. **Conclusions**: PTZ-induced epilepsy causes significant histopathological changes, while cardiac tissue structure is largely preserved in the treatment groups. In our literature review, we did not find any previous studies examining the effects of carbamazepine coated with two different types of nanoparticles on the cardiac morphology in an experimental epilepsy model.

## 1. Introduction

Epilepsy is a common neurological disorder characterised by recurrent seizures and affects approximately 50 million people worldwide [1]. The pathophysiology of epilepsy is complex and multifactorial, involving genetic, structural, and environmental factors [2]. Clinically, epilepsy is defined by the occurrence of at least two unprovoked seizures at least 24 h apart [3]. Symptoms of seizures can vary widely, ranging from brief losses of consciousness to severe convulsions, and can significantly affect the quality of life of affected individuals [4].

The cardiac side effects of antiepileptic drugs in epilepsy are a major concern. The cardiac conduction system can be affected by antiepileptic drugs such as carbamazepine and phenytoin, which are sodium channel blockers [5]. Known for its efficacy in the treatment of various seizure disorders, carbamazepine (CBZ) is a widely used antiepileptic drug. However, its narrow therapeutic index complicates its administration and requires careful monitoring of patient drug levels to avoid toxicity and ensure efficacy [6]. The relationship between epilepsy and heart disease has received increasing attention in recent years, in particular because of the implications for the management and outcomes of patients with epilepsy. Research suggests that people with epilepsy have a higher prevalence of cardiovascular comorbidities, which may worsen their condition and increase mortality [7,8]. Cardiovascular health is a growing area of interest, especially in relation to the long-term effects of antiepileptic drugs like CBZ [9,10].

Cardiotoxicity from carbamazepine may include sinus node dysfunction, atrioven-tricular block, and bradycardia-tachycardia syndrome [11]. Although the mechanism of these effects is not fully understood, the cardiac effects of carbamazepine need to be investigated in animal models.

Studies show an increased risk of cardiac arrhythmias and other cardiovascular complications in patients with epilepsy treated with enzyme-inducing antiepileptic drugs, particularly carbamazepine [12]. Recent advances in nanotechnology have led to the development of carbamazepine carbon nanodot functionalised silver nanoparticles (CBZ-CNDs@AgNPs), which show promise for improving the ability to detect and monitor CBZ levels in biological systems [13]. The unique properties of silver nanoparticles (AgNPs), such as their high surface area and their ability to enhance Raman scattering, make them suitable candidates for the development of sensitive biosensors for the monitoring of therapeutic drugs [13]. Metal–organic frameworks (MOF-5), such as carbon nanodots functionalised silver nanoparticles, are nanoporous materials. In recent years, MOF-5 have come to the forefront as drug delivery systems. MOF-5 are recognised as potential drug carriers for the treatment of cardiovascular diseases such as pulmonary arterial hypertension [14]. CBZ loaded metal–organic frameworks (CNDs@MOF-5) may allow the further improvement of material properties by controlling structural defects in MOF-5.

In our study, we aimed to investigate the effect of carbamazepine on the morphology of the heart using CBZ-CNDs@AgNPs and CNDs@MOF-5 nanoparticles. No data on the effect of CBZ coated with CBZ-CNDs@AgNPs and CNDs@MOF-5 nanoparticles on the heart were found in the literature. Our study may be the first study in the literature with this aspect.

## 2. Materials and Methods

### 2.1. Animal Model and Ethical Approval

Ethical approval for all animal experiments was granted by the Ordu University Local Ethics Committee for Animal Experiments (Decision no. 2023/18, dated 24 May 2023). The study involved male Wistar/albino rats. These rats were aged between 8 and 12 weeks and weighed between 250 and 300 g. The rats were tested at the Ordu Experimental Animals Centre, where the temperature was set at 21 °C and the rats were kept in a 12 h light/dark cycle. They had unlimited access to pelleted food and water.

Seizure responses to PTZ were evaluated according to Racine’s scoring system. The recordings were analysed in a blinded manner to determine seizure stage, latency, and duration. Rats were observed for 30 min. Complete kindling was considered to be achieved when the animal reached stage 4 or 5 seizures following three consecutive PTZ injections.

The Racine scale was used to grade seizure severity, ranging from 0 (no response) to 6 (death), with intermediate stages defined as follows: 1—myoclonic jerks, 2—back tremors, 3—clonic seizures, 4—tonic–clonic seizures, and 5—generalised tonic–clonic seizures [15].

### 2.2. Experimental Design

The rats were randomly divided into ten groups (n = 7 in each group) as follows. G1 (control group): No treatment (baseline control group). G2 (Untreated PTZ group): Rats in this group received 35 mg/kg PTZ intraperitoneally every two days. G3 (Carbamazepine 25 mg/kg, AgNP form): Animals were administered 25 mg/kg carbamazepine (AgNP form) daily by oral gavage. G4 (Carbamazepine 50 mg/kg): Rats received 50 mg/kg carbamazepine (AgNP form) daily by oral gavage. G5 (PTZ + Carbamazepine 25 mg/kg): PTZ was injected intraperitoneally at 35 mg/kg every two days and 25 mg/kg carbamazepine (AgNP form) was given orally each day. G6 (PTZ + Carbamazepine 50 mg/kg): PTZ was administered intraperitoneally at a dose of 35 mg/kg every two days, while 50 mg/kg carbamazepine (AgNP form) was simultaneously given daily by oral gavage. G7 (Carbamazepine 25 mg/kg, MOF-5 form): Rats received 25 mg/kg carbamazepine (MOF-5 nanoparticle form) daily via oral gavage. G8 (Carbamazepine 50 mg/kg, MOF-5 form): Animals were treated with 50 mg/kg carbamazepine (MOF-5 nanoparticle form) administered orally by gavage once a day. G9 (PTZ + Carbamazepine 25 mg/kg, MOF-5 form): PTZ was injected intraperitoneally at 35 mg/kg every two days, while 25 mg/kg carbamazepine (MOF-5 nanoparticle form) was administered daily by oral gavage. G10 (PTZ + Carbamazepine 50 mg/kg, MOF-5 form): Rats received PTZ intraperitoneally at a dose of 35 mg/kg every two days and concurrently 50 mg/kg carbamazepine (MOF-5 nanoparticle form) was administered orally once daily.

Chemicals: Carbamazepine (C4024, Sigma-Aldrich, St. Louis, MO, USA); pentylenetetrazole (PTZ) (P6500, Sigma-Aldrich, St. Louis, MO, USA).

**Figure 1:**
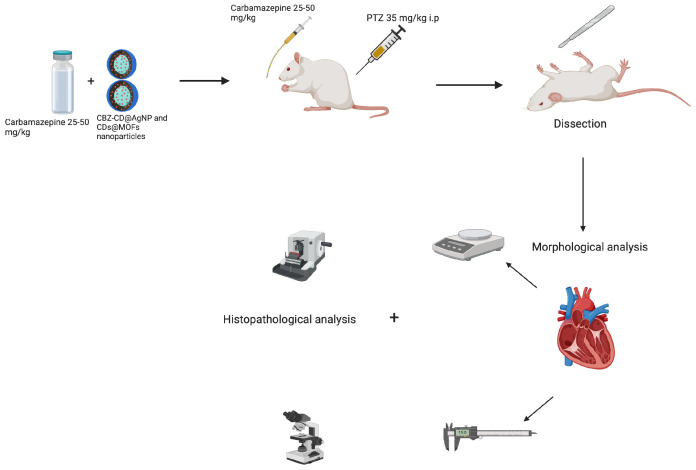
Summary of our experimental work: Nanoparticular coating of carbamazepine, PTZ injection, heart dissection, morphological measurements, and histopathological analysis.

The graphical summary of the study is shown in Figure 1.

### 2.3. Histological Analysis

After euthanasia, the heart samples were immersed in 10% neutral buffered formalin. This was performed promptly. The samples were left in the formalin for 24 h. Then, the tissues were dehydrated using a series of ethanol solutions with increasing concentrations (70%, 80%, 95%, and 100%), cleared in xylene, and embedded in paraffin. Serial sections of 5 µm thickness were obtained using a rotary microtome and stained with hematoxylin and eosin (H&E) for general histopathological assessment. Microscopic images were captured. This was performed using an Olympus BX53 microscope (Olympus Corporation, Tokyo, Japan).

### 2.4. Biochemical Analysis

Interleukin-6 (IL-6), catalase (CAT), and oxidative stress index (OSI) levels were determined by spectrophotometry. Blood plasma samples obtained from rats were used for biochemical analyses. IL-6, CAT, and IL-6 levels were evaluated in plasma. Rat CAT (Sunred Bio, Cat. No: 201-11-5106) and Rat IL-6 (Sunred Bio, Cat. No: 201-11-1036) levels were determined according to the manufacturer’s protocol. Concentrations were determined at 450 nm with ELISA reader. Results are given in ng/mL (or nmol/mL) for IL-6, CAT, and OSI.

### 2.5. The Synthesis of CBZ-CD@AgNP, Carbamazepine-MOF-5, and SEM Image Analysis

Carbamazepine carbon nanodot-functionalized silver nanoparticles (CBZ-CNDs@AgNPs) were synthesised in two main steps. First, carbon nanodots were prepared via a hydrothermal method at 180 °C for 3 h. The obtained carbon nanodots were then used as a reducing and stabilising agent in the ascorbic acid–silver nitrate medium, forming the CBZ-CNDs@AgNPs through in situ reduction.

For the carbamazepine-loaded MOF-5, the MOF-5 structure was synthesised in the presence of carbamazepine, allowing drug molecules to be incorporated during the crystal growth process.

All nanoparticle samples were characterised using TEM, UV–Vis, FT-IR, EDX, and elemental mapping analyses. The TEM images revealed uniformly distributed nanosized particles, while FT-IR confirmed the successful functionalization and drug incorporation. UV–Vis spectra indicated typical surface plasmon resonance for silver nanoparticles and characteristic absorption of carbamazepine. EDX and mapping analyses verified the homogeneous distribution of C, O, N, and Ag (and Zn in MOF-5), confirming the successful synthesis and compositional integrity of both nanostructures [16].

### 2.6. Statistical Analysis

All statistical analyses were conducted using GraphPad Prism software (version 9.0; GraphPad Software Inc., San Diego, CA, USA). Differences among the groups were assessed with a one-way analysis of variance (ANOVA). The Kolmogorov–Smirnov test was used to check whether the data followed a normal distribution. After confirming normality, post hoc comparisons were performed. These used Tukey’s multiple comparison test. The level of confidence was set at 95%. A *p*-value below 0.05 was considered to be statistically significant.

## 3. Results

### 3.1. PTZ-Induced Seizures in Rats

The control group of rats did not exhibit any unusual behaviour after saline injection. Their feeding habits and water consumption continued as normal. In the PTZ + CBZ 50 mg carbon dot group, head shaking, facial twitching, staring, and scratching were observed in rats from the third injection after PTZ administration. After the fourth injection, one rat died due to status epilepticus. In the PTZ + 25 mg MOF-5 group, after the fifth injection and eighth injection, two rats died due to status epilepticus. In the PTZ group, two rats died after the fifth injection and two rats died after the seventh injection. In order to maintain the total number of rats, the rats that died were replaced with new ones. After the thirteenth injection, generalised tonic–clonic seizures and spring muscle spasms were observed in rats. The PTZ kindling-induced epilepsy model was successfully established in rats. Seizures of the rats were observed between approximately 2 and 12 min after injection.

### 3.2. Histological Findings of Heart Tissue with H&E

**Figure 2:**
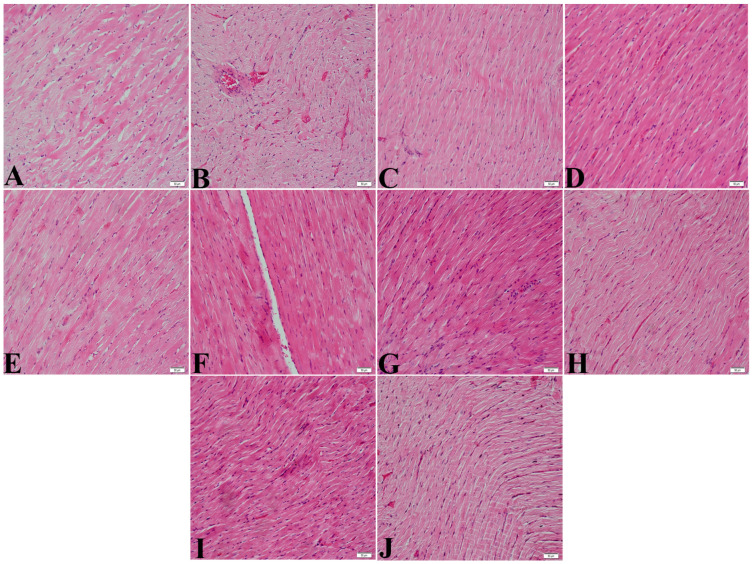
Hematoxylin and eosin staining images of heart tissue. (**A**) Group 1, (**B**) Group 2, (**C**) Group 3, (**D**) Group 4, (**E**) Group 5, (**F**) Group 6, (**G**) Group 7, (**H**) Group 8, (**I**) Group 9, (**J**) Group 10. Image magnification X200.

The heart images of each group after haematoxylin–eosin staining are shown in Figure 2. When the heart tissues were evaluated histopathologically in all groups, it was observed that cells with pyknotic nuclei and haemorrhagic areas were increased in the heart images, especially in the PTZ group which had only epilepsy. Histologically normal heart cells and heart tissue were observed in the other groups.

### 3.3. Morphological Findings of the Heart

**Figure 3:**
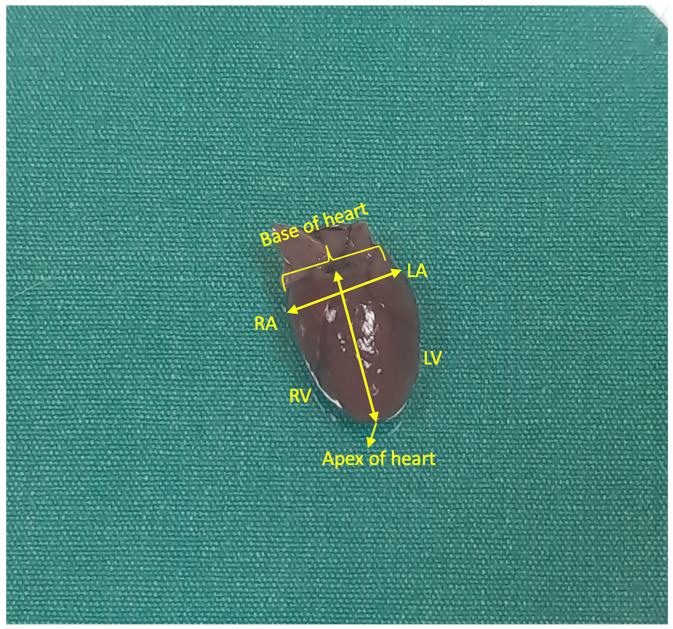
Morphological appearance of the rat heart (RA: right atrium, RV: right ventricle, LA: left atrium, LV: left ventricle).

After the experiment, the rats were dissected under anaesthesia. The chest wall was opened by cutting the costae from the lateral regions. The heart was examined morphologically, and, after measurements, the large vessels at the base of the heart were dissected and removed from the chest cavity. After the structures around the heart were cleaned, the weight of the heart was measured with a precision balance. In addition to the weight of the heart, the distance between the atria and the distance between the apex of the heart and the base of the heart were measured (Figure 3). All these measurements were performed by the same person using the same callipers and precision balance.

The mean heart weight in the control group (G1) was 1.49 ± 0.08 g, while it was 1.08 ± 0.12 g and 1.09 ± 0.18 g in the CBZ-CNDs@AgNPs 25 mg/kg and CBZ-CNDs@AgNPs 50 mg/kg groups, respectively. In the CNDs@MOF-5 25 mg/kg and CNDs@MOF-5 50 mg/kg groups given PTZ, it was 1.10 ± 0.11 g and 1.20 ± 0.09 g, respectively. There was a statistical difference between the groups (Table 1). Statistical analysis demonstrated significant differences in heart weight values among the groups (*p* < 0.05). Post hoc comparisons showed that the heart weights of animals in G1 (control) differed significantly from those in G4, G5, G6, G8, G9, and G10. Additional differences were detected between G2 and G5/G9, and between G3 and G5/G9. These results suggest that high-dose carbamazepine and nanoparticle formulations lead to a marked reduction in heart weight compared with control and low-dose groups.

Regarding IL-6 levels, post hoc analysis revealed significant pairwise differences (*p* < 0.05) between G2 (PTZ) and G7, G8, G9, and G10, as well as between G4 (CBZ 50 mg/kg) and G10 (PTZ + CBZ MOF-5 50 mg/kg), and between G2 and G9. These findings indicate a pronounced anti-inflammatory effect associated with nanoparticle-based CBZ formulations.

The distance between the atria was below 10 mm only in the PTZ + CBZ 50 mg/kg and PTZ+ CNDs@MOF-5 25 mg/kg groups. The distance between the apex of the heart and the base of the heart was the lowest in the CNDs@MOF-5 25 mg/kg and CNDs@MOF-5 50 mg/kg groups.

### 3.4. Biochemistry

**Figure 4:**
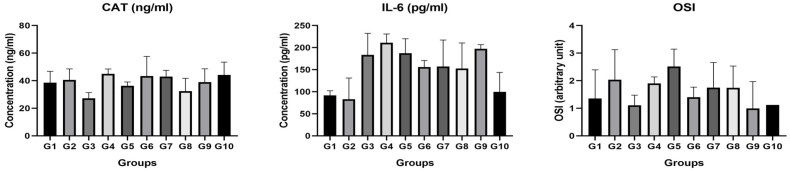
CAT, IL-6, and OSI activities.

When the biochemical results were analysed, no statistical difference was found between the groups in CAT activity (Figure 4). When IL-6 activity was analysed, there was a statistical difference between the groups (*p* = 0.0006). There is a statistical difference between G2 and G9 (*p* = 0.031). There is a difference between G4 and G10 (*p* = 0.039). There was no statistical difference between the groups in OSI activity (*p* > 0.05).

### 3.5. The Synthesis of CBZ-CNDs@AgNPs, Carbamazepine-MOF-5, and SEM Image Analysis

The synthesis of the relevant chemicals is available in our previously published work [16].

## 4. Discussion

Epilepsy is a neurological disorder that affects mental and physical health worldwide [17]. Studies show that PTZ-induced models are a reliable way to study the causes of epilepsy because they replicate human seizure states and responses to antiepileptic medications. The recurrence of seizures has a profound impact on the quality of life of people with epilepsy and leads to various misconceptions in society [18]. Carbamazepine (CBZ), used in the treatment of epilepsy, is an antiepileptic drug particularly effective in seizure control [19]. However, given the effects of carbamazepine on the heart and potential cardiovascular risks, it is not known whether this treatment method may cause side effects [20].

In recent years, nanomedical applications, especially nanoparticle-based systems, offer promising solutions to improve the targeted delivery of drugs [21,22]. Likewise, nanoparticle systems such as metal–organic frameworks (MOFs) have emerged as a promising approach to overcome such limitations [23,24,25]. The high surface area, tunable porosity, and biocompatibility of MOFs facilitate the targeted delivery of drugs [26]. Carbamazepine applications formulated with MOF nanoparticles show significant changes in the control of seizures, especially during epileptogenesis. Studies have shown that rats receiving MOF nanoparticles in combination with carbamazepine have a significant reduction in seizure frequency and severity [27]. These results suggest that the efficacy of carbamazepine can be increased and side effects can be reduced by using MOF systems [28]. Our study highlights that silver nanoparticles are effectively functionalized with carbamazepine carbon nanodots and potentially improve their physicochemical and biological properties for various applications.

Carbon dots are fluorescent nanoparticles, usually smaller than 10 nm, known for their membrane permeability and resistance to environmental influences [29]. These nanoparticles allow carbamazepine to reach the target tissue more efficiently. Carbon dots have a low toxicity profile on cells and offer high biocompatibility, making them attractive for drug delivery systems [30]. In recent years, significant attention has been given to the application of carbon dots (CDs) in the field of biomedical research as a way to reduce the adverse effects of various drugs, including carbamazepine, on heart tissue. Typically characterised as carbon nanoparticles smaller than 10 nm, carbon dots exhibit remarkable properties such as low toxicity, high biocompatibility, and desirable optical characteristics, making them suitable candidates for therapeutic applications and biosensor technologies [31,32]. Studies indicate that the application of carbamazepine coated with carbon dots to cardiac tissue alters its pharmacological effects on the heart and has the potential to reduce cardiovascular side effects [32]. In our study, EDX spectrum analysis and SEM mapping results show that CBZ-CNDs@AgNPs have been successfully synthesised and their main components are homogeneously distributed. In particular, the confirmation of carbon, oxygen, nitrogen, and zinc elements by SEM mapping supports the structural integrity of the CBZ-CNDs@AgNP coating. This indicates that the CBZ-CNDs@AgNPs’ structure is an effective carrier system in increasing bioavailability and enabling the drug to reach the target site.

During histopathological evaluations, marked changes were observed in the heart tissues of carbamazepine-treated rats. The adverse effects of carbamazepine on the heart include structural changes such as necrosis and fibrosis in myocardial cells [33]. In our study, when the heart tissues were evaluated histopathologically in all groups, it was observed that pyknotic nucleated cells and haemorrhagic areas were increased in the heart images, especially in the PTZ group with epilepsy only. Histologically normal cardiac cells and cardiac tissue were observed in the other groups. Pyknotic nuclei are typically associated with apoptosis, a process of programmed cell death. In the context of epilepsy, this suggests that PTZ-induced repeated seizures may cause neuronal damage and death. This increase in pyknotic nuclei in the untreated PTZ group is consistent with findings from other studies examining the cellular effects of seizures in animal models.

C-reactive protein (CRP) and interleukin-6 (IL-6) levels, which are indicators of oxidative stress and inflammation, play an important role in the evaluation of the effects on cardiac health as a result of carbamazepine administration in male rats used as epilepsy models [33]. In the literature, carbamazepine treatment has been reported to increase oxidative stress in the heart. The oxidative stress index (OSI) is recognised as an indicator of the oxidative damage experienced by cells, which may threaten the integrity of the cardiac tissue [33]. In our study, there was no statistical difference between the groups in CAT and OSI activities. When IL-6 activity was analysed, there was a statistical difference between the groups (*p* = 0.0006). IL-6 is considered to be a part of the inflammatory response and its increase is associated with increased risk of heart diseases. There is a statistical difference between the PTZ group and PTZ + Carbamazapine 25 mg/kg (MOF-5) group (*p* < 0.05). There is also a significant difference between the Carbamazepine 50 mg/kg group and the PTZ + Carbamazepine 50 mg/kg group (*p* < 0.05). IL-6 is a key pro-inflammatory cytokine associated with cardiac stress and tissue injury. The markedly elevated IL-6 levels in the PTZ group indicate systemic inflammation and myocardial stress induced by seizures. In contrast, the reduced IL-6 levels in the nanoparticle-treated groups—particularly in PTZ + CBZ-MOF-5 25 mg/kg and PTZ + CBZ-MOF-5 50 mg/kg—suggest that the nanoparticle-based carbamazepine formulations exert anti-inflammatory and cardioprotective effects. This decrease implies the attenuation of seizure-related inflammatory cascades, improvement of oxidative balance, and a lower likelihood of cardiac injury. Functional cardiac assessments (e.g., ECG, echocardiography, cardiac enzyme levels) were not performed in our study. Therefore, the functional correlations of the observed morphological preservation remain unclear and should be addressed in future studies.

## 5. Conclusions

Based on the present findings, carbamazepine formulations coated with silver-coated carbon dot and MOF-5 nanoparticles appear to have potential biomedical applications that may contribute to future strategies in epilepsy treatment. In our study, the lowest heart weights were observed in the G5 and G6 groups and there was a statistical difference between all experimental groups. When the distance between the atria and the distance between the apex of the heart and the base of heart were analysed, there was no difference between the groups. While intense pyknotic cells were observed in the heart tissue in the G2 group, it was determined that this was not the case in the other groups. The potential benefits of MOF-5 and carbon dot nanoparticles may shed light on the research on minimising the side effects of carbamazepine, but more scientific data are needed in this regard.

## Figures and Tables

**Table 1:** Morphological data of the heart according to experimental groups.

Groups	Heart Weights (g)	Distance Between Atrium (mm)	Distance Between the Apex of the Heart and the Base of the Heart (mm)
G1	1.49 ± 0.08	11.5 ± 1.2	16.5 ± 1.7
G2	1.27 ± 0.08	11.5 ± 1.0	16.0 ± 1.6
G3	1.30 ± 0.13	11.4 ± 0.8	17.0 ± 1.2
G4	1.14 ± 0.07	11.2 ± 1.8	15.2 ± 1.5
G5	1.08 ± 0.12	10.5 ± 1.9	14.7 ± 2.2
G6	1.09 ± 0.18	9.7 ± 3.4	16.7 ± 1.8
G7	1.27 ± 0.17	11.0 ± 0.8	16.0 ± 1.4
G8	1.10 ± 0.20	10.0 ± 1.2	14.1 ± 2.7
G9	1.10 ± 0.11	9.3 ± 0.5	13.0 ± 1.0
G10	1.20 ± 0.09	10.0 ± 1.0	14.3 ± 0.5
*p*	0.002 *	0.06	0.4

* Statistically significant difference (*p* < 0.05).

## Data Availability

The data that support the findings of this study are available on request from the corresponding author. The data are not publicly available due to privacy or ethical restrictions.
